# Effect of Limestone Powder Mixing Methods on the Performance of Mass Concrete

**DOI:** 10.3390/ma17030617

**Published:** 2024-01-27

**Authors:** Lele Zhao, Tingshu He, Mengdie Niu, Xiulong Chang, Lei Wang, Yan Wang

**Affiliations:** 1 School of Materials Science and Engineering, Xi’an University of Architecture and Technology, Xi’an 710055, China; zhaolele@xauat.edu.cn (L.Z.); changxl2023@163.com (X.C.); 2 Post-Doctoral Mobile Stations of Civil Engineering, Xi’an University of Architecture and Technology, Xi’an 710055, China; 3 Shaanxi Hongqi Hui Shi Construction Production Co., Ltd., Xianyang 710055, China; wl97282024@163.com (L.W.); wangyanyan83@163.com (Y.W.)

**Keywords:** limestone powder, mass concrete, hydration temperature, compressive strength, constrained expansion rate, impermeability

## Abstract

Using limestone powder (LP), the by-product of manufactured sand, to replace part of fly ash (FA) or manufactured sand could not only turn waste into treasure and decrease the price of concrete, but could also enhance the performance of concrete and reduce environmental pollution. However, the impact of various LP incorporation methods on the performance of mass concrete was inconsistent. In this paper, the effects of LP on the workability, compressive strength, constrained expansion rate, hydration temperature and impermeability of mass concrete were studied by replacing FA or manufactured sand alone and replacing FA and manufactured sand simultaneously. The results showed that the impact of LP on the performance of mass concrete was equal when it replaced FA alone and FA and manufactured sand at the same time. When the replacement amount was 20%, the workability, expansibility and early strength of concrete were improved, but the later strength and impermeability were slightly reduced. The workability, compressive strength, expansibility and impermeability of mass concrete were improved when manufactured sand was replaced alone, and the optimal dosage was 10%. The LP, moreover, reduced the hydration temperature peak of concrete in three kinds of mixing methods, but the temperature peak appeared earlier. At lower dosages, LP optimized pore structure and promoted the early hydration of cement through filler effects and nucleation effects. When LP replaced manufactured sand, the microstructure of concrete was more dense, so the replacement of manufactured sand had a better effect on the improvement of concrete properties. A reference value for the use of LP in mass concrete is provided in this study.

## 1. Introduction

A great number of mass concrete structures have been constructed as infrastructural construction has developed rapidly. Mineral additives like FA and slag powder are widely used for reducing cement content, reducing the heat of hydration and enhancing the mechanical properties and durability of mass concrete. However, the supply of high-quality mineral additives is limited, so it is important to realize the sustainable development of mass concrete to seek suitable alternative materials and reduce the amount of cement [1]. As a by-product of the production of manufactured sand, LP has a wide range of raw material sources and features, including filling effects, diluting effects and nucleating effects [2,3,4,5], which can not only optimize the properties of concrete, but also lower the adiabatic temperature rise of concrete. However, the current disposal measures of LP are mainly through accumulation and landfills [6,7], and its application in mass concrete can not only realize the transformation of waste into treasure and solve the problem of the shortage of admixture supply, but can also reduce carbon emissions in the environment. The carbon emissions of concrete were reduced by approximately 36% when a supplementary cementitious material of 50% cement mass was used [8].

LP reduces the water requirement of paste through a filler effect, thereby improving the workability of fresh concrete [9,10]. Vuk et al. [11] found that for every 5% increase in LP content, the water requirement for the normal consistency of paste decreased by 0.5% on average. However, Rizwan et al. [12] believed that excessive incorporation of LP would reduce the fluidity of concrete. In addition, Li et al. [13] showed that compared with the group without LP, when LP of 10% cement weight was used, the slump loss of concrete was slightly reduced and the early strength was significantly improved. Meanwhile, the fluidity and compressive strength of ultra-high-performance concrete were increased by adding a small amount of LP [14]. As a supplementary cementitious material, on the one hand, the nucleation site provided by LP can promote the early hydration of cement, which will increase the compressive strength of concrete [15,16,17]. On the other hand, LP will dilute cement clinker, resulting in a reduction in the total quantity of products of hydration, which will decrease the mechanical properties of concrete [18]. Vance et al. [19] reported that 21% of the 28 d strength of the mortar was lost if the amount of cement replaced by LP was 20%. The replacement of fine aggregates with LP resulted in a significant improvement in the compressive strength of concrete, which was attributed to the filler effect of LP [20]. Vijayalakshmi et al. [21] confirmed that LP content within 15% had no adverse effect on concrete strength. Nikbin et al. [22] showed that as the LP content was raised to 100% from 25%, the compressive strength of W/C 0.6 and 0.47 improved by 20% and 38%, respectively. In addition, Bayesteh et al. [23] showed that mortar obtained the best compressive strength when the LP content was 20%.

Different from ordinary concrete, due to the large size of the structure of mass concrete, the heat released by cement hydration accumulates and is not easy to be transmitted, resulting in a large temperature difference between the internal and external portions of concrete, which produces temperature stress and leads to structural cracking. Some scholars believe that nanocellulose has great potential for strengthening concrete structures [24]. Some studies have found that the cumulative heat of hydration of the paste declines significantly as the LP content rises [25,26]. However, Kim et al. [27] demonstrated that adding LP shortened the introductory stage of the hydration heat curve and raised the peak in the heat flow. Han et al. [28] concluded that adding LP enlarged the exothermic rate and cumulative heat of hydration in the early stage of mortar, especially for fine LP. Therefore, the impact of LP on the heat of hydration release is unclear, and further experimental research is needed. In addition, expansion agents are usually added to compensate for the shrinkage of concrete and reduce its cracking risk in mass concrete projects. Guo et al. [29] believed that the expansion performance of the calcium sulfoaluminate expansion agent (UEA) mainly occurred before 14 days, while the MgO expansion agent played an expansion role in the later period. The combined use of UEA and MgO expansion agents could improve the interfacial transition zone of concrete and reduce cracks caused by shrinkage [30]. In addition, calcium carboaluminate generated by the reaction between tricalcium aluminate and LP inhibited the conversion of ettringite (AFt) to calcium monosulfoaluminate and stabilized AFt formed in the early stage [5]. Therefore, whether adding LP is beneficial to the development of the expansion performance of expansion agents remains to be explored.

Mass concrete (such as that in foundations and rafts) is mainly used underground. Many harmful substances infiltrate concrete along pores and cracks and affect its performance. Therefore, impermeability is a significant index of concrete durability [31]. Wang et al. [20] found that when the particle size of LP was small and the content was low, the pore diameter of concrete was optimized and the porosity was reduced due to its filler effect, thus improving the chloride permeability resistance of concrete. However, when the particle size of LP was larger and the dosage was higher, the dilution effect increased the porosity of concrete, and thus reduced the chloride permeability resistance of concrete. It was found that adding nanocellulose can improve the impermeability of concrete [32]. Li et al. [33] showed that when LP increased from 0% to 12%, the chloride electric flux of cement-based materials decreased by 46.7%, and the water permeable depth decreased by 51.8%. The chloride diffusion coefficient of concrete is higher as LP increases, especially if the LP content exceeds 15% [34,35,36]. Bonavetti et al. [37] noted that when the LP content was raised to 20% from 10%, the chloride diffusion coefficient increased to 114% from 43%. The addition of nanocellulose can also improve the impermeability of concrete.

To sum up, the current research on LP mainly focuses on the effect of LP alone replacing cement or fine aggregates on the performance of ordinary concrete. However, the contribution of LP to the hydration temperature and the expansion property of expansion agents in mass concrete is still unclear. Therefore, in this paper, cement, FA, LP and expansion agents are used as cementation materials to prepare mass concrete. The relationships between LP and the workability, compressive strength, hydration temperature, constrained expansion rate and impermeability of mass concrete are studied through different incorporation methods. Meanwhile, the mechanism of influence is revealed by microscopic analysis. This paper aims to offer a rationale for the adoption of LP in mass concrete.

## 2. Materials and Methods

### 2.1. Materials and Mix Ratios

**Figure 1:**
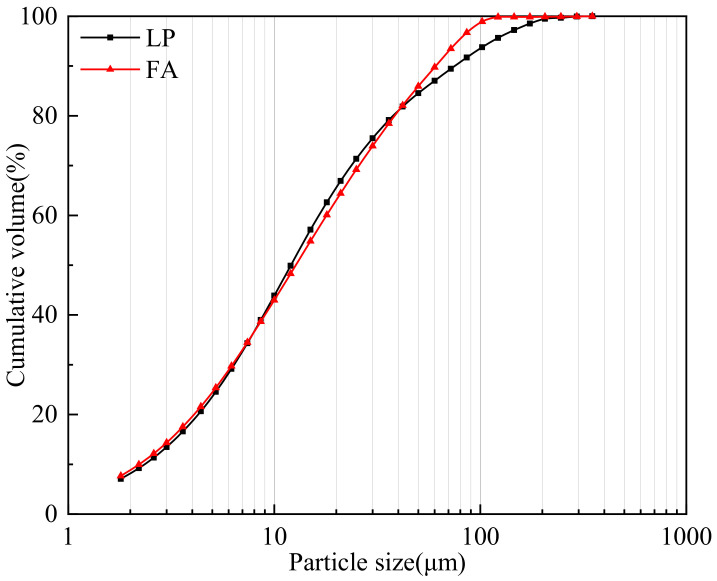
Particle size distribution of LP and FA.

P·O 42.5 ordinary Portland cement according to Chinese national standard GB175-2007 [38] was used in this study, with 7 d and 28 d compressive strengths of 43.6 MPa and 53.2 MPa, respectively. Cement, Class II FA, a UEA expansion agent and LP were used in all concrete mixes. All raw materials were provided by Shaanxi Hongqi Hui Shi Building Products Co., Ltd., Xianyang, China. Table 1 provides the chemical composition and physical properties of the raw materials. Manufactured sand with a specific gravity of 2720 kg/m^3^, a particle diameter of under 4.75 mm and a fineness modulus of 2.8 was selected as the fine aggregate. The coarse aggregate was crushed stone with a specific gravity of 2740 kg/m^3^ and continuous gradation of 5~25 mm. A superplasticizer of the polycarboxylate type (PS) was used with a solid content of 8% and a water-reducing rate of 12%. Figure 1 shows the LP and FA particle size distributions determined by a Laser Particle Size Analyzer (HELOS-RODOS, SYMPATEC, Rostock, Germany). Mass concrete with a design strength of 30 MPa was selected as the target of study, and the concrete mix proportions are shown in Table 2. JZ: LP was not used. LF: LP replaced FA only. LFS: LP was sifted by a 75 μm square-hole sieve, less than 75 μm (about 75%) to replace FA, greater than 75 μm (about 25%) to replace manufactured sand. LS: LP only replaced manufactured sand.

### 2.2. Test Methods

The method for determining the workability of fresh concrete was in line with Chinese standard GB/T 50080-2016 [39]. The values of slump and slump flow at 0 h and 1 h were tested using a slump cylinder to evaluate the fluidity.

Concrete cube specimens with a side length of 100 mm were prepared in accordance with Chinese standard GB/T 50081-2019 [40] for compressive strength measurement. Specimens cured for 24 h in a molding chamber at 20 °C were removed from the mold and then transferred to a standard curing chamber (20 ± 2 °C, RH over 95%). The compressive strength was measured at 7, 28, 60 and 90 days, respectively.

The impermeability of concrete, which mainly includes water impermeability and chloride impermeability, was measured by Chinese standard GB/T 50082-2009 [41]. The water penetration depth, which was used to characterize the water impermeability, was tested using circular platform specimens of Φ175 mm × Φ185 mm × 150 mm, with six specimens per group. The electric flux measured by rapid chloride penetration test was used to characterize the impermeability of chloride. Cylindrical specimens of Φ100 mm × 50 mm were used in the experiment, with 3 specimens per group.

Three prismatic specimens of 100 mm × 100 mm × 300 mm were prepared for each proportion, conforming to Chinese standard GB/T 23439-2017 [42], to determine the constrained expansion of concrete. For the first 14 days, the specimens were immersed in a constant temperature tank with a temperature of (20 ± 2) °C for curing. After 14 days, the specimens were placed in a room with a fixed temperature and humidity (20 ± 2 °C, 60 ± 5% RH). The lengths of specimens at 1, 3, 7, 14, 21, 28, 35, 42, 60 and 90 days were measured, respectively.

An EX4000 multi-channel temperature tester(EX4000, Dongguan, China) was used to measure and plot the hydration temperature curve of cement paste for 72 h at (25 ± 2) °C [43]. Table 2 shows the mixture proportions of JZ, LF (20% and 60% of LP), LFS (20% and 60% of LP) and LS (10% and 20% of LP), in which sand, stone and superplasticizers were removed and the proportion of other materials remained unchanged, that were used to prepare paste samples.

In order to perform scanning electron microscopy (SEM, CLARA, TESCAN, Brno, Czech Republic) tests, 5 mm × 5 mm × 2 mm mortar samples without coarse aggregates were drilled from the center of concrete specimens cured to the tested age. Before testing, the samples were soaked in absolute ethanol for 48 h to terminate hydration and dried in a 50 °C oven for 15 h. In order to perform X-ray diffraction (XRD, D8 ADVANCE, Bruker, Karlsruhe, Germany) analysis and thermogravimetric analysis (TGA, STA 449 F5, NETZSCH, Bavaria, Germany), the cement paste specimens made according to the concrete mix were cured to the specific age, and a little central part of the specimens was immersed in anhydrous ethanol for 48 h and cured for 15 h in a 50 °C oven. The dried specimens were ground to powder. The XRD tests were carried out in the range of 5°~90°, and TGA tests were conducted using a process of heating from 25 °C to 900 °C with a constant velocity of 10 °C/min.

## 3. Results and Discussion

### 3.1. Workability

The fluidity of fresh concrete is usually characterized by slump or slump flow, and the influence of LP content on fluidity is given in Table 3.

From Table 3, it is observed that the influence law of LP content on concrete workability was similar in the LF group and the LFS group. As the replacement quantity of LP increased, the slump and slump flow of the concrete increased and then declined. The slump and slump flow of 20–60% LP were higher than those of the JZ group at 0 h. Although the values decreased at 1 h, the figures for the 20% and 40% LP dosages were still higher than those for JZ. It is obvious that the slump and slump flow at 0 h and 1 h were the largest when the dosage of LP was 40%. During the experiment, it was found that the cohesiveness and water retention of fresh concrete became significantly worse when the dosage of LP exceeded 60%. When the LP content was 100%, the fresh concrete bled seriously. This is because the fine LP played a filler effect, the water in the paste was displaced and the free water content in the concrete was increased [44]. Compared with the LF group and the LFS group, there were obvious differences in the workability of fresh concrete in the LS group. During the experiment, the water retention of fresh concrete was improved significantly with the increase in LP. However, when the dosage of LP was more than 10%, the cohesiveness of concrete was remarkably increased, and the slump and slump flow loss at 1 h increased. Compared to the values of slump and slump flow at 0 h, the 1 h values were reduced by 7–50% and 14–33%, respectively, when the LP content was 5–25%. The reason for this phenomenon is the larger MB value of LP and the higher clay content in the fine material, which adsorbs a larger amount of water and PS [45]. In addition, compared to FA, the roughness and angular texture of LP increase the friction between coarse aggregate and cement paste, which increases the specific surface area, water absorption rate and water requirement of the powder [25,46,47,48]. Therefore, keeping the water consumption and PS content constant, as the LP content increased, the cohesiveness of fresh concrete increased, the fluidity deteriorated and the 1 h slump loss increased. To sum up, there is an optimal amount of LP to improve the workability of concrete [49], and the influence mechanism of LP is different with various mixing methods.

### 3.2. Compressive Strength

**Figure 2:**
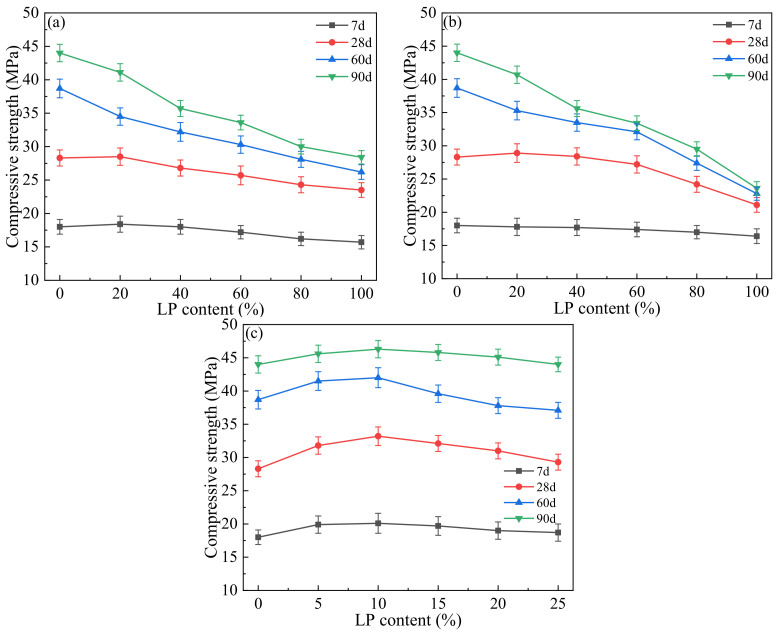
Influence of LP content on compressive strength: (**a**) LF, (**b**) LFS, (**c**) LS.

Figure 2 illustrates the impact of LP content on the compressive strength of concrete.

The greatest change in the compressive strength of the JZ group can be observed in Figure 2a,b, where the strength increased by 36.7% and 55.5% at 60 d and 90 d, respectively, compared to that at 28 d. After adding LP, the 7 d strength of concrete in the LF group and LFS group had different change rules. As the content of LP increased, the 7 d strength of the LF group initially rose and then fell, reaching its highest strength at 20% content, while the 7 d strength of the LFS group gradually decreased. The strength of concrete increased obviously from 7 d to 28 d, but there was little difference between different dosages at the same curing age. At 28 d, 60 d and 90 d, the same change pattern of compressive strength in the LF and LFS groups can be easily observed. As the LP content was added, the strength at 28 d initially rose and gradually declined, while the strength at 60 d and 90 d gradually decreased, which is significantly different from that at 28 d. The reason is that LP exhibited a dilution effect and reduced the compressive strength, which has also been found in many studies [10,50]. Furthermore, LP has a lower activity than FA, and the replacement of FA by LP has a greater weakening effect on later hydration than the filler effect and nucleation effect of LP, which also leads to a decrease in the compressive strength of concrete. In particular, if LP replacement is greater than 20% and the curing age is 90 d, a slight increase in concrete strength is observed. This suggests that the replacement of FA by LP is not conducive to the development of strength in the later stages of concrete, and that the larger the dosage of LP, the greater the loss of strength. From Figure 2c, the compressive strength showed a tendency of increasing and then decreasing as the amount of LP substitution increased in the LS group. The maximum strength was obtained at the addition of 10% LP, which is 1.6%, 10.3%, 9.1% and 6.4% higher than that of the JZ group at 7 d, 28 d, 60 d and 90 d, respectively. At 7 d, compared with JZ, the concrete strength of the LS group had no obvious change, which is similar to that in the LF group and LFS group. Compared with 28 d, the strength of the LS group increased significantly at 60 d and 90 d, and even with 25% of the content, the strength was similar to that of the JZ group. From 60 d to 90 d, the strength of concrete increased by 5~7 MPa, and the strength and strength change law of the LS group were significantly different from those of the LF and LFS groups. This is because of the filler effect of LP, which optimizes the internal pore size of the concrete, and the pyroclastic effect of FA, which enhances the later strength development of the concrete. Therefore, the appropriate amount of LP is beneficial to raise the compressive strength of concrete. At the same time, the strength at 60 d and 90 d in practical engineering deserves attention.

### 3.3. Constrained Expansion Rate

**Figure 3:**
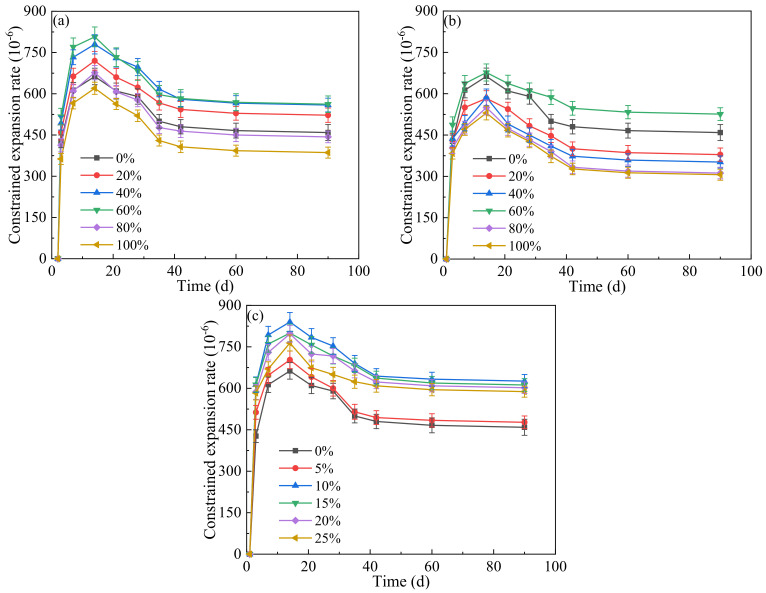
Influence of LP content on restrained expansion rate: (**a**) LF, (**b**) LFS, (**c**) LS.

The influence of LP content on the constrained expansion rate of concrete at different ages is shown in Figure 3.

The effects of the three alternative methods of LP use on the constrained expansion rate were similar. The constrained expansion rate increased significantly in the first 7 d and slowly from 7 d to 14 d, reaching the maximum at 14 d. The nucleating action of LP promoted cement hydration at an early stage, and the rapid reaction of the expansion agent with calcium hydroxide (CH), one of the hydration products of cement, under the early immersion curing condition generated a large amount of expansive substance Aft [51,52]. The constrained expansion rate gradually decreased from 14 d to 42 d and did not change after 42 d. The reason is that the air humidity was low, and the expansion agent could not be fully hydrated depending on the internal moisture of the concrete, resulting in insufficient Aft generation [52]. In addition, the pozzolanic activity of FA requires CH to stimulate it, and the CH content in the concrete decreased, resulting in the weakening of the expansion effect of the later expansion agent [53]. As seen in Figure 3a,b, the constrained expansion rate of each age in the LF and LFS groups reached its maximum value at 60% LP content. In the LF group, it was observed that the values of the constrained expansion rate at the same age were greater than those in the JZ group when the LP dosage was less than 60%. The curves at the 40% and 60% LP dosages were almost coincident and higher than those at 20%. In the LFS group, the constrained expansion rate was greater than that in the JZ group only when the LP dosage was 60%. In addition, when the same dosage of LP was used, the constrained expansion rates of the LFS group were all smaller than those of the LF group. The transformation process of Aft to calcium monosulfoaluminate was inhibited by the hydrated calcium carboaluminate produced by the reaction between LP and the aluminum phase of cement, and the stabilization of Aft promoted an enlargement of the whole volume of hydrated products [5,25,54]. However, as the water–binder ratio and PS remained unchanged, the porosity of concrete rose with the increasing amount of LP due to the small water requirement ratio of LP [26]. The hydration product Aft of the expansion agent was not enough to fill the pores and it was difficult to achieve the ideal expansion effect [55]. Therefore, the constrained expansion rate firstly rose and later reduced as the LP dosage increased. In Figure 3c, it is found that the constrained expansion rate of the LS group was greater than that of the JZ group, and the maximum value was reached when the LP content was 10%. The cohesiveness of fresh concrete increased obviously as the LP dosage increased, and the concrete assumed a state of water shortage. When the water consumption was insufficient, the expansion agent preferentially competed for water due to its faster hydration reaction than that of cement, thus generating a large amount of Aft to produce the ideal amount of expansion [55]. Compared with the LF group and the LFS group, it can be clearly observed that the LS group had the best expansion performance. It can be inferred that the constrained expansion rate was strongly associated with the amount of LP and the method of incorporation. The constrained expansion rate was promoted by adding the appropriate amount of LP, but over a certain amount, the LP had a negative effect on the constrained expansion rate.

### 3.4. Hydration Temperature

**Figure 4:**
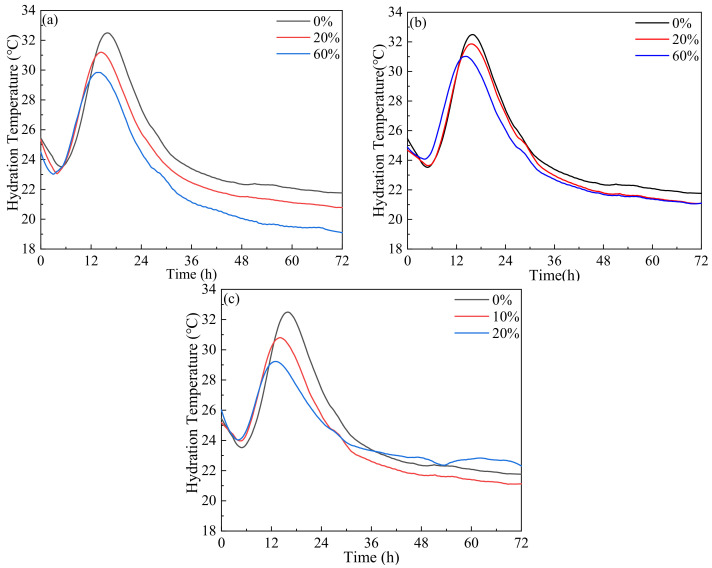
Influence of LP content on hydration temperature; (**a**) LF, (**b**) LFS, (**c**) LS.

The influence of different LP contents on the hydration temperature of paste is shown in Figure 4. It is obvious that the three incorporation methods had similar effects on the hydration temperature. The hydration temperature of paste from 0 to 6 h gradually decreased, increased sharply from 6 to 18 h, decreased sharply from 18 to 36 h and decreased gently after 36 h. The hydration temperature peak appeared from 12 to 18 h. As the amount of LP increased, the hydration temperature peak of paste declined obviously, indicating that the addition of LP was beneficial to reduce the hydration temperature. However, the time of the hydration temperature’s peak gradually advanced. Due to the nucleation effect of LP, the introductory stage of cement hydration was shortened and the acceleration period was advanced [2,56]. It can be seen from Figure 4a that in the LF group, compared with the JZ group, when 20% and 60% LP were added, the hydration temperature peaks were reduced by 1.3 °C and 2.7 °C and the time of the hydration temperature peak was advanced by 1.5 h and 2.0 h, respectively. As seen in Figure 4b, compared with the JZ group, the hydration temperature peak in the LFS group was reduced by 0.7 °C and 1.5 °C, respectively, when LP was added in 20% and 60% amounts, and the time of the hydration temperature peak was advanced by 0.2 h and 1.6 h, respectively. As explained in Figure 4c, in group LS, when 10% and 20% LP were added, the hydration temperature peaks were reduced by 1.7 °C and 3.3 °C, and the time of the hydration temperature peak was advanced by 1.8 h and 2.9 h, respectively. When compared to the LF group and LFS group, the hydration temperature of the LS group was significantly lower, but the hydration temperature peak appeared significantly earlier.

### 3.5. Impermeability

**Figure 5:**
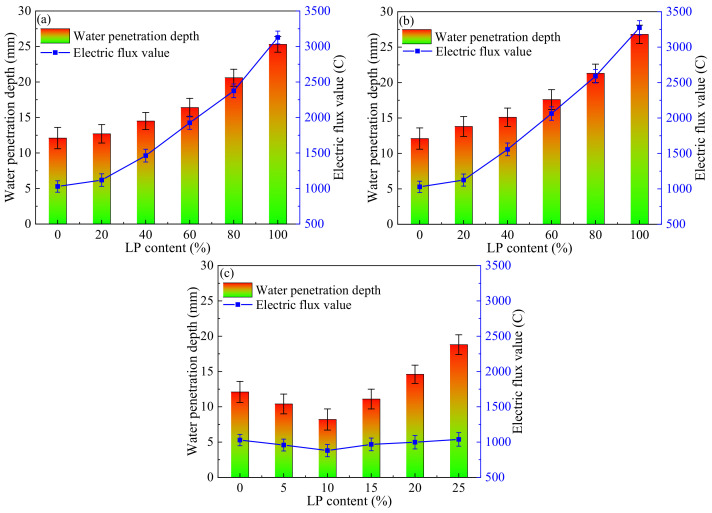
Influence of LP content on impermeability: (**a**) LF, (**b**) LFS, (**c**) LS.

The influence of LP content on the water penetration depth and chloride electric flux value of concrete after 28 days of curing can be seen in Figure 5.

From Figure 5a,b, the influence of LP content on concrete impermeability was similar in the LF group and LFS group. The water penetration depth and the electric flux value of concrete increased in proportion with the LP content, and the values of the JZ group were the smallest, which were 12.1 mm and 1028.37 C, respectively. Compared with the LFS group, the water penetration depth and electric flux value of concrete in the LF group were smaller. Compared with the JZ group, in the LF group and LFS group, the water penetration depth increased by 1.7~14.7 mm and 0.6~13.2 mm, and the electric flux value increased by 95.76~2244.92 C and 90.19~2098.10 C, respectively, with the addition of 20~100%. This shows that as the content of LP increased, the impermeability of concrete in the LF and LFS groups became worse. As a supplementary cementitious material, a higher LP content increases the porosity and leads to a decrease in the impermeability of concrete [20]. However, from Figure 5c we can see that the influence of LP content on concrete impermeability in the LS group was significantly different from that in the LF group and the LFS group. The water penetration depth and electric flux value of concrete increased initially and decreased later as the LP content increased. Especially at a content of 10% LP, the water penetration depth and electric flux value were the smallest, which were reduced by 3.8 mm and 150.88 C, respectively, compared with those in the JZ group. One possible reason is that LP filling between aggregates can refine the internal pore structure and improve the impermeability of concrete [33]. In addition, the water penetration depth and electric flux value of the LS group were significantly reduced compared with those of the LF and LFS groups. This indicated that the best impermeability is obtained by replacing manufactured sand with LP.

### 3.6. SEM

**Figure 6:**
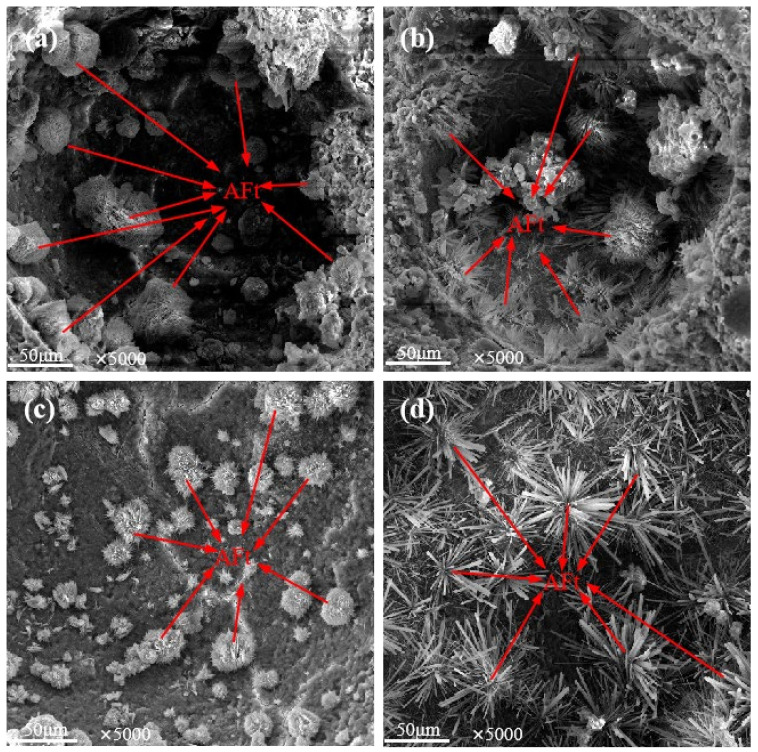
SEM images of AFt in pores: (**a**) JZ0, (**b**) LF20, (**c**) LFS20, (**d**) LS10.

**Figure 7:**
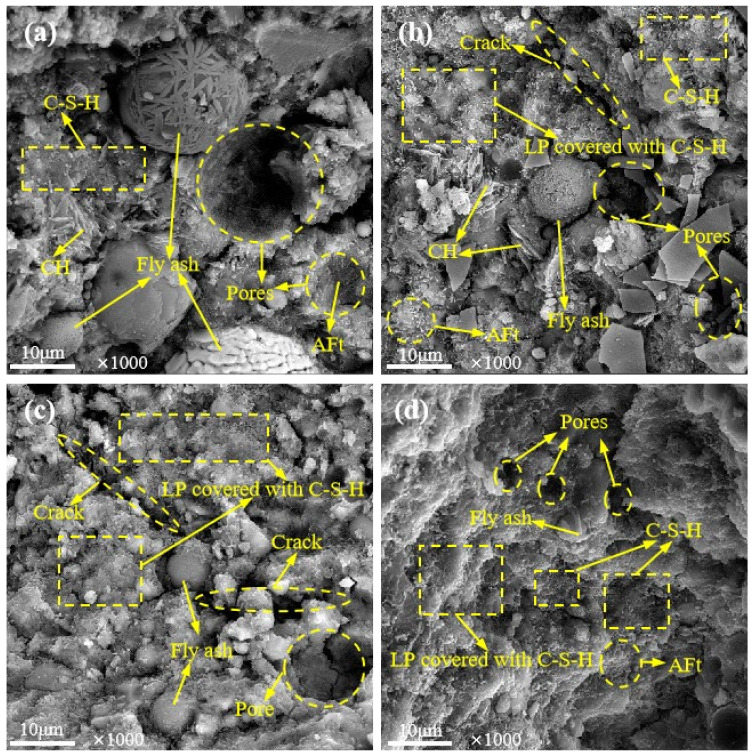
SEM images of microstructure: (**a**) JZ0, (**b**) LF20, (**c**) LFS20, (**d**) LS10.

To further investigate how LP affects the concrete microstructure, SEM analysis was carried out on the concrete samples of the JZ, LF, LFS and LS groups with a curing age of 60 d and LP contents of 0%, 20%, 20% and 10%, respectively (denoted as JZ0, LF20, LFS20 and LS10, respectively, the same below), as shown in Figure 6 and Figure 7.

The microstructure of Aft in concrete pores is illustrated in Figure 6, which clearly shows that a large number of AFt crystals grow outwards along the inner wall of the pores in clusters or fibrous rods. The comparison in Figure 6a,c shows that the AFt crystals of JZ0 and LFS20 were interwoven into yarn balls and filled the internal pores of the concrete, but the AFt size of LFS20 was smaller than that of JZ0. It is clear from Figure 6b,d that the AFt crystals of LF20 and LS10 grew outward along the inner wall of the pore and crisscrossed to form a complex network structure that occupied the inner space of the pore. Compared with JZ0 and LFS20, the AFt crystals of LF20 and LS10 were abundant and larger in size, and the spatial network structure was complex, which is more conducive to improving the strength and durability of concrete.

The internal microstructure of concrete is shown in Figure 7. It is shown that the presence of AFt, C-S-H gels and unhydrated FA particles in the concrete. CH was also found in JZ0 and LF20, as shown in Figure 7a,b. In a comparison of Figure 7a–c, it was found that there were large pores in JZ0, LF20 and LFS20, and the pores in JZ0 were filled by Aft. Compared with JZ0, micro-cracks existed in both LF20 and LFS20, and the cracks in LFS20 were wider and more numerous. This corresponds to results of the low compressive strength and poor impermeability of LFS20 in the macroscopic properties. In addition, compared with JZ0, C-S-H gel covering the surface of LP particles was observed to grow in LF20, LFS20 and LS10. This is because the Ca and O atoms in the crystal structure of LP are similar to the planar configurations in C-S-H gel, which can provide a good nucleation growth site for C-S-H gel [3]. At the same time, LP particles, C-S-H gel and needle-rod AFt overlap each other and fill the internal space of the concrete. In particular, a structure with smaller pore volumes and denser matrixes can be clearly observed in LS10. This is consistent with the result that this concrete had the highest strength and the best impermeability.

### 3.7. XRD

**Figure 8:**
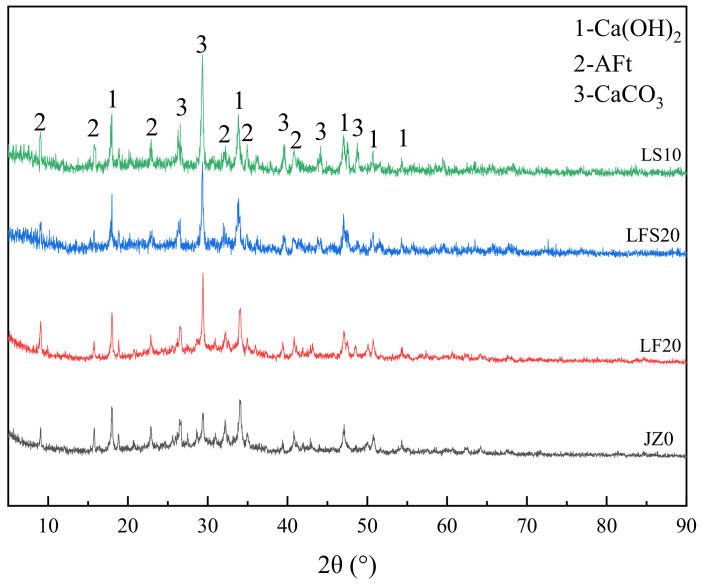
XRD pattern of cement paste at 7 d.

The XRD patterns of JZ0, LF20, LFS20 and LS10 at 7 d are shown in Figure 8. As the amount of LP in LF20, LFS20 and LS10 increased gradually, the peak intensity of calcium carbonate in the XRD image gradually increases, and the order is LF20 < LFS20 < LS10. The presence of calcium carbonate in JZ0 may have been caused by carbonization. LF20 and JZ0 had similar CH peak intensities and LFS20 and LS10 had little difference in their CH peak intensities, but compared with JZ0, the CH peak intensity increased. This indicates that more CH was generated by cement hydration in the LFS20 and LS10 samples. Compared with LFS20, the AFt peaks in JZ0, LF20 and LS10 were more obvious, and the AFt peaks in LF20 and LS10 were stronger than those in JZ0. This indicates that AFt was abundant in LF20 and LS10, which also confirms the results observed in the SEM image. This is attributed to the nucleation action of LP, which accelerates the initial hydration process of cement and provides abundant CH for the expansion agent, thus promoting the hydration of the expansion agent [52]. The macroscopic properties show that the concrete strength and constrained expansion rate of LF20 and LS10 were increased.

### 3.8. TGA

**Figure 9:**
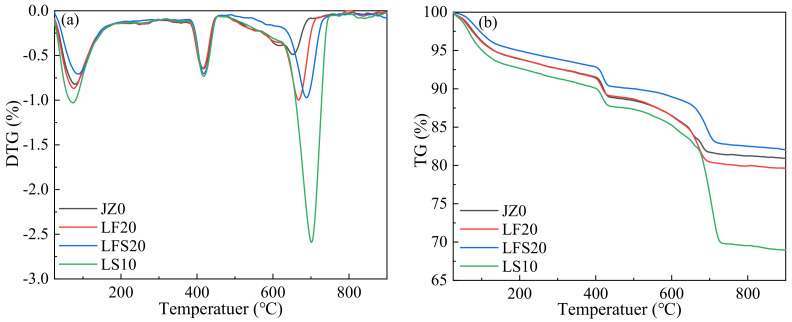
TGA images of cement paste at 7 d: (**a**) DTG curve, (**b**) TG curve.

The TGA test results of the cement paste samples of JZ0, LF20, LFS20 and LS10 with curing ages of 7 d are shown in Figure 9.

In the DTG curve at 80~100 °C, the first peak was related to the evaporative loss of water, free and chemically combined, from C-S-H gels and AFt, which corresponds to the mass loss in the TG curves at 80–100 °C. The second peak near 400 °C was due to the dehydroxylation of CH, which corresponds to the mass loss at 400~500 °C in the TG curve. The AFt peak in LF20 was slightly larger than that in JZ0, and the CH peak almost coincides with both. This indicates that the AFt and CH contents in LF20 and JZ0 were similar. This is because in the early stage, FA and LP had small hydration activities, which are mainly filled, and the hydration component was only cement, resulting in a similar amount of hydration products. However, the AFt peak in LFS20 was smaller than that in JZ0, and the CH peak was greater than that in JZ0, which suggests that the CH content in LFS20 was higher and the AFt content was lower than those in JZ0. The reason is that as an auxiliary cementitious material, the diluting effect of LP leads to a reduction in the total quantity of hydration products [19]. At the same time, it is further explained why the strength and restrained expansion rate of LF20 were higher than those of JZ0, but LFS20 was smaller than JZ0 at 7 d. The peaks in AFt and CH in LS10 were increased compared with those in JZ0, LF20 and LFS20, indicating that the AFt and CH contents in LS10 were the highest. These results agree with those observed in the XRD pattern. It is concluded that LP not only had a filler effect, but also had a nucleation effect in the LS group, which promoted the early hydration of cement. It played an active role in improving the compressive strength, the constrained expansion rate and the impermeability. It was also reflected in the macroscopic properties that the concrete strength and constrained expansion rate of LS10 were greater than those of JZ0, LF20 and LFS20. In addition, compared with JZ0, the third peaks observed at about 700 °C for LF20, LFS20 and LS10 were due to the decomposition of calcium carbonate, which caused a mass loss of 700~800 °C in the TG curve.

## 4. Conclusions

In this paper, the effects of different replacement methods of LP on the workability, compressive strength, constrained expansion rate, hydration temperature and impermeability of mass concrete are studied. Combined with the microscopic test results, the following conclusions are summarized:

(1) Adding an appropriate amount of LP increases the workability of concrete. The optimum dosage of LP for replacing FA is 40% and the best workability of concrete is obtained when 10% of the manufactured sand is substituted with LP.
(2) The compressive strength of concrete shows no significant change in the early stages (7 d and 28 d) and gradually decreases in later periods (60 d and 90 d) with an increasing dosage of LP replacing FA. The peak compressive strength is reached when 10% of the amount of manufactured sand is substituted with LP.
(3) The constrained expansion rate initially grows and then shrinks as the amount of LP increases. The hydration temperature gradually decreases as the addition of LP increases, but the highest temperature peak appears earlier.
(4) The impermeability of concrete gradually deteriorates as the amount of LP replacing FA increases. The best impermeability of concrete is obtained when 10% of the total amount of manufactured sand is replaced with LP.
(5) The lower dosage of LP refines the pore structures of aggregates and promotes the early hydration of cement through filling and nucleation effects, which improves the performance of concrete.

## 5. Future Research Direction

In the future, the influence of the type, particle size and absorbability of stone powder on the performance of mass concrete with different strength grades can be studied, especially in terms of adiabatic temperature rises and compensation shrinkage. In addition, it is necessary to establish the quantitative relationship between limestone powder content and carbon emissions.

## Figures and Tables

**Table 1:** Chemical composition and physical properties of raw materials.

Composition/Properties		Cement	FA	UEA	LP
SiO_2_ (%)		21.89	49.62	25.35	0.69
Al_2_O_3_ (%)		4.46	17.52	15.80	0.36
Fe_2_O_3_ (%)		2.16	6.63	0.80	0.14
MgO (%)		1.38	1.15	0.77	0.37
CaO (%)		66.74	7.59	26.30	54.79
Na_2_O (%)		0.20	1.09	0.10	-
K_2_O (%)		0.59	2.29	0.50	0.08
SO_3_ (%)		1.12	1.04	26.80	0.01
Water demand ratio (%)		100	97	-	93
Methylene Blue adsorption (MB, g/kg)		-	-	-	1.25
Compressive strength ratio (%)	7 d	100	69	94	67
	28 d	100	71	100	69

**Table 2:** Mixture proportions of concrete (kg/m^3^).

No.	LP Content	Cement	FA	LP	UEA	Sand	Stone	Water	PS
JZ	0%	195	135	0	36.6	856.7	1005.7	165	6.59
LF	20%	195	108	27	36.6	856.7	1005.7	165	6.59
	40%	195	81	54	36.6	856.7	1005.7	165	6.59
	60%	195	54	81	36.6	856.7	1005.7	165	6.59
	80%	195	27	108	36.6	856.7	1005.7	165	6.59
	100%	195	0	135	36.6	856.7	1005.7	165	6.59
LFS	20%	195	108	36	36.6	847.7	1005.7	165	6.59
	40%	195	81	72	36.6	838.7	1005.7	165	6.59
	60%	195	54	108	36.6	829.7	1005.7	165	6.59
	80%	195	27	114	36.6	820.7	1005.7	165	6.59
	100%	195	0	180	36.6	811.7	1005.7	165	6.59
LS	5%	195	135	43	36.6	813.7	1005.7	165	6.59
	10%	195	135	86	36.6	770.7	1005.7	165	6.59
	15%	195	135	129	36.6	727.7	1005.7	165	6.59
	20%	195	135	172	36.6	684.7	1005.7	165	6.59
	25%	195	135	215	36.6	641.7	1005.7	165	6.59

**Table 3:** Influence of LP content on fluidity.

No.	LP Content (%)	Slump		Slump Flow	
		0 h (mm)	1 h (mm)	0 h (mm)	1 h (mm)
JZ	0	210	205	575	535
LF	20	215	205	610	580
	40	225	210	645	585
	60	215	200	600	485
	80	210	195	585	440
	100	205	200	560	430
LFS	20	215	205	600	575
	40	225	210	620	580
	60	220	205	600	500
	80	215	200	580	450
	100	210	205	570	445
LS	5	210	195	570	500
	10	215	185	555	485
	15	210	160	485	380
	20	210	115	465	340
	25	200	100	440	295

## Data Availability

Data are contained within the article.

## Abbreviations

All abbreviations in this article are decoded as follows:

Abbreviation	Full name
LP	Limestone powder
FA	Fly ash
UEA	Calcium sulfoaluminate expansion agent
AFt	Ettringite
CH	Calcium hydroxide
MB	Methylene blue adsorption
PS	Polycarboxylate superplasticizer
SEM	Scanning electron microscopy
XRD	X-ray diffraction
TGA	Thermogravimetric analysis
